# Editorial: Artificial intelligence in cutaneous lesions: where do we stand and what is next?

**DOI:** 10.3389/fmed.2024.1420152

**Published:** 2024-05-08

**Authors:** Mara Giavina-Bianchi, Justin Ko

**Affiliations:** ^1^Medical Image Research Department, Hospital Israelita Albert Einstein, São Paulo, Brazil; ^2^Clinical Dermatology, School of Medicine, Stanford University, Stanford, CA, United States

**Keywords:** artificial intelligence (AI), cutaneous diseases, dermatology, skin cancer, teledermatology, acne, patient survey, systematic literature review

We have seen, with great interest and enthusiasm, the continued growth in research output detailing the use of Artificial Intelligence (AI) in cutaneous diseases as can be seen in Figure 1, as well as the maturation of content of the research bridging the gap from hype to reality; from pixels to practice (1).

**Figure 1:**
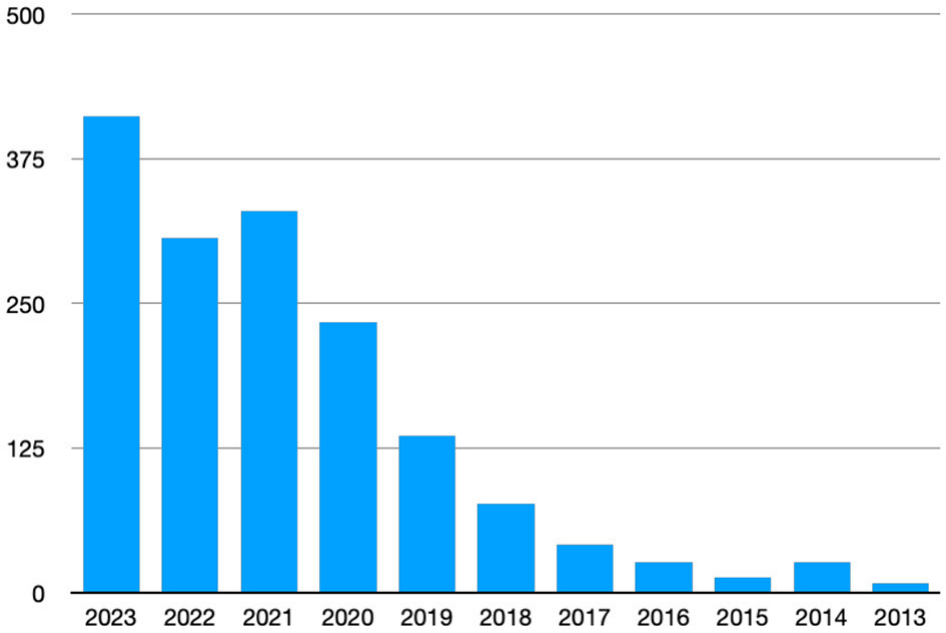
Articles for “artificial intelligence and dermatology” on Pubmed by year.

The body of work spans a broad range, from skin cancer detection (2–4), inflammatory skin diseases (5, 6) surveys with dermatologists (7), patients perspectives (8), among others [(9); Giavina-Bianchi et al.]. While we are starting to see the initial glimpses of what clinical practice augmented and supported by AI capabilities might look like we do not yet have tools used regularly by dermatologists, other clinicians, or patients in daily practice. Why is this? Where do we stand now? What is next in this field? To try answer these questions, this special Research Topic solicited articles and resulted in 10 manuscripts from teams diverse in geographic representation as well as topic were accepted and published to shed light on these questions.

In setting the stage to answer the question “where are we now?”, Furriel et al. provided a systematic review of papers specifically on AI as applied to the detection, classification, and assessment of skin cancer images in the clinical setting. Their rigorous methodology identified 18 studies that encompassed a diversity of approaches in skin cancer detection, as well as significant differences in dataset size. They highlight the areas of convergence and divergence in the work and approaches to this topic, including more focused binary tools vs. broader approaches with multiclass output.

Two papers provide additional reflections on the state of the art as well as starting to answer the question “where are we going?”. Omiye et al. provided a broad overview of artificial intelligence (AI), as applied to dermatology with a primary focus on methodology, AI applications for various skin diseases, limitations, and future opportunities. They reviewed the current image-based models, highlighted the challenges facing widespread adoption and the future of AI in evolving the paradigm of large language, and multi-modal models.

Wei et al. discuss clinical applications including novel areas outside of visual assessment, as well as new methodological approaches like federated learning, multimodal learning, and new model architectures like vision transformers. The confluence of technological breakthroughs along with the breadth of clinical applications means that there will be opportunity for Research Topic on AI applied to dermatology for many years to come!

A set of four articles in the topic series focused on and highlight AI in real-world practice. They cover different aspects of pioneering endeavor in UK that is bringing these AI tools and capabilities into clinical practice with measurable benefit: from the model development to patient perceptions around the use of technology in aiding clinical decision-making. First, Marsden et al. had a goal to help improve the triage and management of suspicious skin lesions, using AI-based Digital Health Technology (DERM-003). This was a prospective, multi-center study that aimed to demonstrate the effectiveness of an AI as a Medical Device (AIaMD) to identify Squamous Cell Carcinoma, Basal Cell Carcinoma, pre-malignant and benign lesions from dermoscopic images of suspicious skin lesions. They found that the AIaMD AUROC varied from 0.85 to 0.89, demonstrating the potential to support the timely diagnosis of malignant and premalignant skin lesions.

Second, they aimed to implement the above AI solution, and safely reduce referral rates. Their objective was to demonstrate that the AIaMD had a higher rate of correctly classifying lesions that did not need to be referred for biopsy or urgent face-to-face dermatologist review, compared to teledermatology standard of care (SoC), maintaining the same sensitivity to detect malignancy. Their results showed a potential to reduce the burden of unnecessary referrals when used as part of a teledermatology service Marsden et al..

Third, patients recruited in this study were asked to complete an online questionnaire to evaluate their views regarding use of AIaMD in the skin cancer pathway by Kawsar et al. The majority of respondents felt confident in computers being used to help doctors diagnose and formulate management plans and as a support tool for general practitioners when assessing skin lesions and had no issues on their photographs being taken with a mobile phone device.

Lastly, Thomas et al. analyzed the real-world performance of the above medical device (AIaMD) tool for skin lesion assessment. They assessed the DERM deployment within skin cancer pathways at two National Health Service hospitals (UK) in 2 versions, which demonstrated very high sensitivity for detecting melanoma or malignancy, in-line with sensitivity targets and pre-marketing authorization research, reducing the caseload for hospital specialists.

The work of MB and team highlights an emerging important aspect of bringing AI capabilities into the real world—that of explainability and interaction with the clinician. This demonstrated the current state and variability between different models of saliency visualization that impacted clinician acceptance and preference. There is much to be done in the real of human/computer interface, and this work shows the nuance and importance of evaluating seemingly simple concepts like how we visualize and show data and information to clinicians (Giavina-Bianchi et al.).

Two additional papers represent progress and innovative approaches—Shavlokhova et al. explore the feasibility of leveraging advances in text-to-image generation capabilities in service of generating synthetic dermoscopic images of disease. While the results show that there is promise in preliminary aspects to this approach, it remains to be seen whether current state gaps in realism can be closed, and whether synthetic data may hold utility in supplementing or augmenting real data (Shavlokhova et al.).

Li et al. tackle a real world clinical use case of training and validating the ability of an algorithm to replicate human acne severity grading, demonstrating the utility of AI capabilities to use cases outside of skin lesion assessment and beyond classification/diagnosis tasks. The potential role for these efforts in creating efficiencies and fostering improved consistency in clinical assessment is on display, though begs the question of whether at this point clinician labeling as gold standard is the true gold standard (Li et al.).

This set of articles makes clear that we have traversed a significant distance from the initial hype around AI in dermatology toward an intimate understanding of what it takes to translate possibility to practice and patient impact.

## Author contributions

MG-B: Writing—original draft, Writing—review & editing. JK: Writing—original draft, Writing—review & editing.
